# 3D-printed device for efficient packing of semisolid samples in 3.2 mm rotors used in cryoprobe systems

**DOI:** 10.1002/mrc.70010

**Published:** 2025-07-10

**Authors:** Andrea Gelardo, Gustavo A. Titaux-Delgado

**Affiliations:** 1 Instituto de Química Física “Blas Cabrera”, Consejo Superior de Investigaciones Científicas (IQF-CSIC), Serrano 119, 28006 Madrid, Spain

**Keywords:** solid-state NMR, cryoprobe MAS, sample packing, semisolid samples, Packing device

## Abstract

We present a compact, 3D-printed device designed to facilitate the efficient packing of semisolid or highly viscous samples into 3.2 mm rotors compatible with cryogenic solid-state NMR probes. The tool enables sample loading by centrifugation under standard laboratory conditions, significantly improving packing reproducibility and minimizing sample loss. In Contrast to previously reported designs for conventional rotors, this device is optimized for the expanded volume and geometrical constraints of 90 μL rotors used in the Bruker CPMAS cryoprobe. A complementary unloading tool is also described to recover samples or enable rotor reuse. Both tools are compatible with standard benchtop centrifuges and are fully customizable. Their implementation improves sample handling for biological or material samples with limited availability or challenging rheological properties. Open-access 3D design files are provided to support broad adoption and future adaptation to other rotor sizes or sample formats. These devices represent a scalable solution for routine use and may inspire further development of customized tools for challenging sample types.

## Introduction

Solid-state nuclear magnetic resonance (ssNMR) has emerged as an essential tool for the structural and dynamic characterization of systems with high molecular complexity, restricted mobility, or intrinsic heterogeneity [1-4]. Unlike other techniques, ssNMR does not require crystalline samples, is independent of solubility, and is not limited by molecular size, allowing access to a wide range of biological samples and functional materials under conditions close to their native state [5-6]. This versatility has enabled the study of systems ranging from inorganic polymers and extracellular matrix biopolymers to supramolecular complexes such as nucleosomes, membrane proteins in lipid bilayers, amyloid fibers, and bacterial cell walls [7-12].

The development of this technique has been closely linked to the progressive increase in magic-angle spinning (MAS) frequencies, a fundamental strategy for suppressing dipolar interactions and chemical shift anisotropy, which would otherwise lead to significant spectral line broadening [13]. Since the implementation of the first 7 mm and 4 mm rotors capable of reaching spinning speeds of approximately 5 and 10–15 kHz, respectively, the technology has evolved toward smaller-diameter rotors, such as 3.2 mm, 2.5 mm, and 1.3 mm, enabling spinning speeds exceeding 60 kHz [14-15]. Currently, rotors of 0.7 mm and even smaller, such as 0.4 mm, can achieve spinning rates above 150 kHz, driving remarkable improvements in spectral resolution and substantially narrowing the gap between the spectral quality of ssNMR, and that of solution-state NMR [16-17].

Proper sample packing in the rotor is a fundamental aspect of solid-state NMR, as it directly influences magnetic field homogeneity and the quality of the resulting spectra [18]. Achieving precise and uniform filling remains a significant challenge. The small internal volumes of ssNMR rotors limit direct manipulation of the sample, increasing the risk of uneven compaction or void formation factors that can compromise both spinning stability and spectral sensitivity [19]. These difficulties become even more pronounced when working with semisolid or highly viscous samples, where controlled transfer and compaction within the rotor demand delicate handling, and reproducibility depends on well-defined sample hydration and density conditions [20-21].

To address the difficulties associated with packing samples into rotors of various sizes, several strategies have been developed to maximize both efficiency and uniformity of loading. One of the most reliable methods for handling semisolid samples is centrifugation, which facilitates uniform compaction while reducing the occurrence of internal voids [22]. In this context, the design of specialized devices using 3D printing has opened new possibilities for precise sample handling and loading, providing accessible and customizable solutions [22-25]. A notable example is the development of adapters and devices compatible with ultracentrifuges, which enable in situ sedimentation of biomolecules directly within the rotor. This approach increases the effective sample concentration within the active volume and enhances both spinning stability and spectral sensitivity, especially when using small-diameter rotors [26-28].

The ongoing technological progress in solid-state NMR spectroscopy has led to significant hardware developments, among which the implementation of MAS cryogenic probes stands out. Similar to their impact in solution-state NMR, these cryoprobes have marked a turning point in sensitivity [29]. Operating at low temperatures and equipped with optimized detectors, they enable a substantial improvement in signal-to-noise ratio, facilitating the acquisition of high-resolution multidimensional spectra from minimal sample amounts [30-32].

In parallel, the implementation of this technology has been accompanied by the introduction of rotors with specific geometries. While maintaining the nominal outer diameter of 3.2 mm, the rotors used in solid-state cryoprobes differ internally from standard designs. Conventional models typically offer an internal volume of approximately 47 μL, whereas cryoprobe-compatible versions can reach up to 90 μL.

With the increasing use of cryogenic probes in the field of solid-state NMR, the demand for practical and broadly accessible devices to support sample preparation in rotors with specialized geometries has become increasingly apparent. Although several devices have been developed to accommodate a variety of rotor types, there are currently no documented devices specifically designed for the internal geometry of the 3.2 mm rotor used in cryogenic MAS probes.

In this work, we propose the design of two 3D-printed devices: one for efficient sample packing by centrifugation, and another for controlled rotor unloading. Both are compatible with standard 1.5 mL tubes and benchtop centrifuges. These devices will be made available as open-access designs to support their implementation in diverse experimental settings and to contribute to the refinement of practical protocols in solid-state NMR.

## Methods

### 3D Printing of Components

The devices used for sample packing and unloading were designed using Blender [33] exported in STL format, and processed with Lychee slicing software [34]. Fabrication was carried out via stereolithography [35] using an Anycubic Photon Mono X 6K 3D printer (Anycubic Technology Company, Hong Kong, China), with a layer height of 50 μm and an exposure time of 2.5 seconds per layer. Washable resin from Anycubic (Anycubic Technology Company, Hong Kong, China) was used for printing. After printing, the parts were transferred to an Anycubic Wash & Cure station (Anycubic Technology Company, Hong Kong, China), where they were washed with water for 30 minutes to remove residual resin. Finally, the printed components were cured under 405 nm ultraviolet (UV) light for 15 minutes at room temperature to ensure complete polymerization of the material.

### Sample Packing

After obtaining the semisolid sample (which may include amyloid fibers or other aggregates), the sample is first centrifuged at 4,000 × g for 10 minutes to concentrate the material. The resulting pellet was carefully transferred to a 1.5 mL microcentrifuge tube and subjected to a second centrifugation at 10,000 × g for 15 minutes to obtain a compact pellet. After centrifugation, the supernatant was gently removed to avoid disturbing the sediment. A 3.2 mm rotor with a 90 μL volume was placed into the 3D-printed device face up and inserted into a 1.5 mL microcentrifuge tube. Since the material of interest was concentrated at the bottom of the previous tube, the lower section of that tube was carefully cut, allowing the segment containing the pellet to be inserted upside down directly into the filling device. This approach enabled a more efficient transfer of the concentrated material into the rotor, minimizing sample loss and providing better control over compaction. The assembled device was then subjected to a final centrifugation at 20,000 × g for 30 minutes.

### Rotor Unloading

To recover the sample or reuse the rotor after NMR acquisition, an auxiliary unloading device was used. The rotor was placed in an inverted position within the device, such that its open end faced downward. The device is compatible with standard 1.5 mL microcentrifuge tubes. This configuration enabled rotor emptying by centrifugation at 20,000 × g for 20 minutes.

## Results and Discussion

The use of ultracentrifugation-based devices has been extensively documented as an effective strategy for sample packing in MAS rotors, particularly through direct sedimentation of material within the rotor [36-37]. In this study, an alternative approach based on conventional centrifugation at 20,000 × g was employed, which proved sufficient to achieve homogeneous compaction of semisolid samples [38]. This choice enabled the design of a device compatible with standard 1.5 mL microcentrifuge tubes and benchtop centrifuges, facilitating its implementation in typical laboratory environments without compromising packing efficiency or spectral quality.

**Figure 1:**
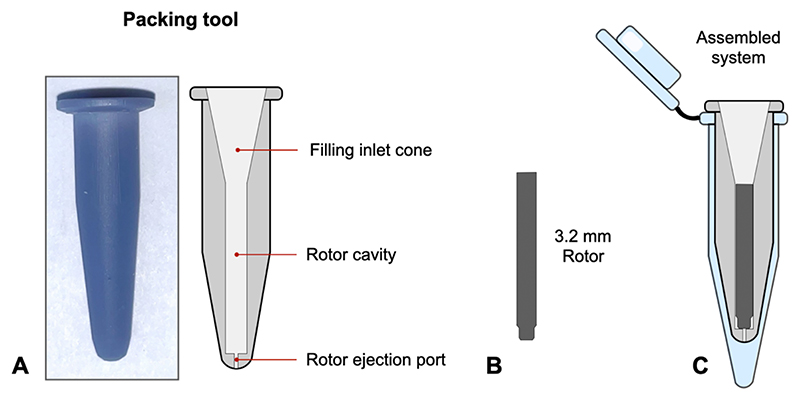
Centrifugation-based packing device for 3.2 mm rotors. A. Schematic representation of the 3D-printed device, highlighting its functional components. B. Standard 3.2 mm rotor used in cryogenic MAS probes. C. Assembled system view, showing the integration of the rotor within the device and its compatibility with standard 1.5 mL microcentrifuge tubes.

Unlike other tools described in the literature that rely on funnel-like structures to guide the sample into the rotor cavity [37], the design proposed here avoids the use of narrow channels prone to clogging. Instead, it features an open conical geometry that directs the material smoothly and continuously into the rotor. This configuration minimizes blockage points and allows for a cleaner and more controlled transfer. The design is shown in Figure 1, which illustrates the integration of the entry cone with the rotor cavity.

During development, it was observed that poor coupling between the structure and the rotor could lead to sample leakage, particularly in the gaps between the printed part and the rotor. To prevent sample loss, the channel diameter was progressively adjusted in 0.01 mm increments until a sufficiently tight fit was achieved, ensuring complete and efficient transfer of the material into the rotor. Due to the high level of precision required, a small hole was also incorporated at the base of the device, allowing the rotor to be gently pushed out using a micropipette tip without causing damage (Figure 1).

The quality of the fit between the device and the rotor largely depends on the geometric accuracy achieved during the printing process. As reported in previous studies (Olson et al., 2024; Amerein et al., 2023), the orientation of the model during stereolithography significantly influences both the resolution and structural stability of parts with defined internal geometries. In this case, it was found that a fully vertical orientation was the most suitable, as it prevented distortions in the central channel where the rotor is inserted, which is critical to avoid sample leakage.

**Figure 2:**
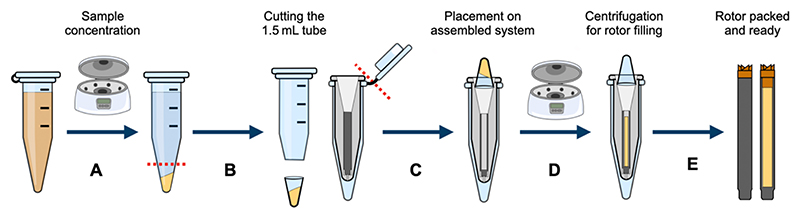
Schematic representation of the sample packing protocol. A. Sample concentration by centrifugation to form a compact pellet. B. Removal of the lower segment of the 1.5 mL tube to expose the pellet. C. Positioning of the tube segment onto the pre-assembled packing device containing the rotor. D. Centrifugation of the system to drive the sample into the rotor cavity. E. Packed rotor ready for use.

Adapting the device design to the standard 1.5 mL tube format enabled a practical sample-packing strategy: directly placing the sample into the device followed by centrifugation. This approach is feasible for certain types of samples, particularly those that are homogeneous and flow easily. However, in practice, many semisolid or viscous samples tend to clog pipette tips, making precise transfer difficult. Although a common solution is to cut off the narrow ends of pipette tips to prevent clogging, this can lead to sample loss if the material is not fully transferred. As an alternative, a more efficient approach was adopted: the sample was first centrifuged in a 1.5 mL tube to form a compact pellet, after which the lower section of the tube containing the concentrated material was carefully cut and inverted onto the filling device. This strategy, inspired by a procedure described by Wang et al. (Nature, 2013) to maximize sample transfer into MAS rotors, significantly reduces handling, minimizes sample loss, and ensures direct transfer of the material into the rotor cavity (Figure 2). Although the proposed centrifugation times worked well for our samples, the compaction (10,000 × g for 15 min) and transfer processes (20,000 × g for 30 min) may vary depending on the properties of each preparation. Therefore, it is advisable to adjust the duration, especially during the final rotor filling step, to ensure efficient packing that guarantees stable and secure rotor spinning. To compensate for potential tilt effects caused by the rotor angle in the centrifuge, the procedure can be repeated after rotating the tube 180 degrees to promote a more uniform distribution.

**Figure 3:**
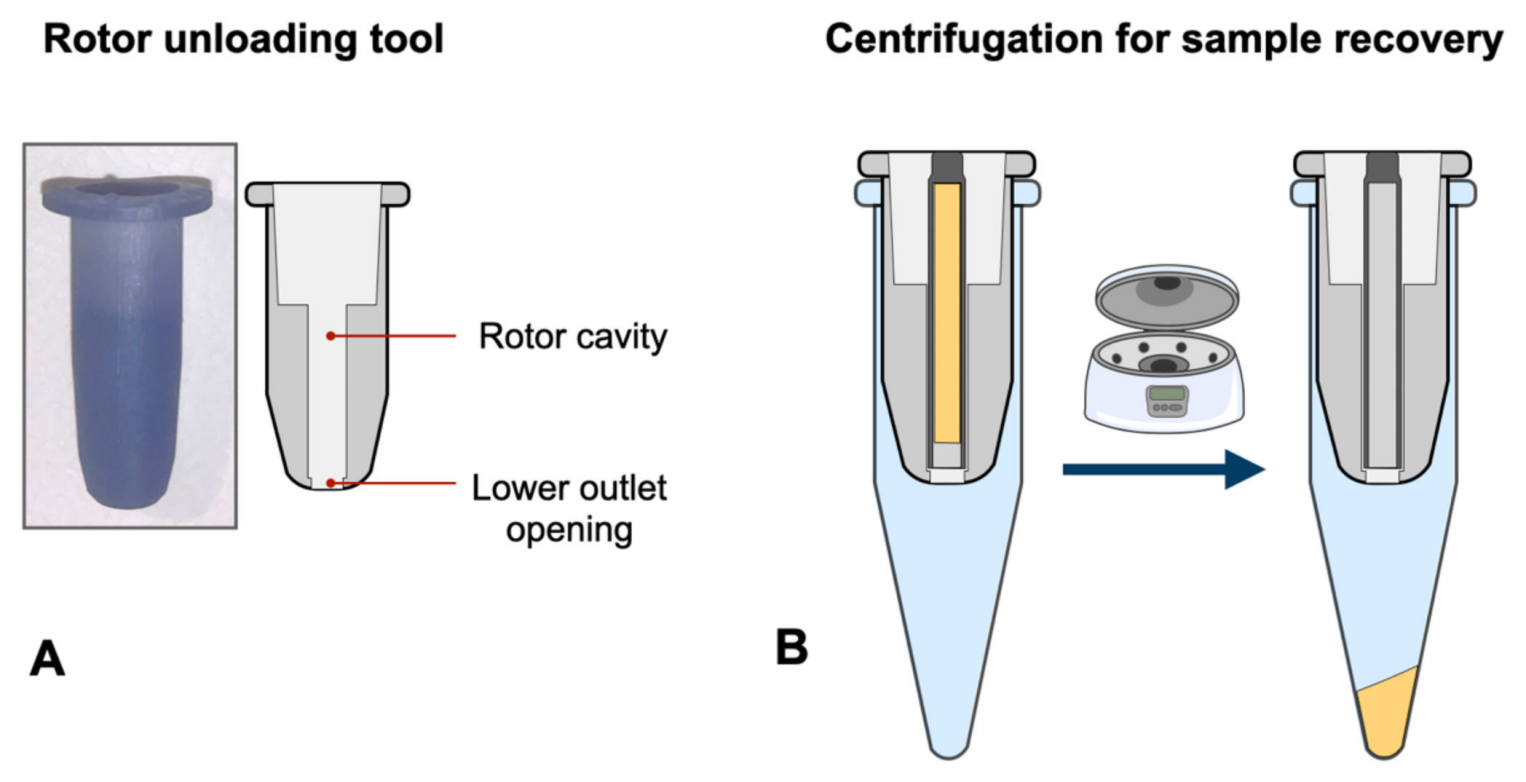
Rotor unloading device. A. Schematic view of the unloading device design, highlighting the rotor cavity and the wide bottom outlet. B. Usage protocol: the rotor is placed in an inverted position within the device and centrifuged to release its contents.

To complete the sample handling system, an auxiliary device was designed for rotor unloading, intended for applications in which the sample needs to be recovered or when the rotor is to be reused without excessive handling. The design remains compatible with standard 1.5 mL tubes and allows the rotor to be placed in an inverted orientation. Unlike the filling device, this model features a wider bottom opening to facilitate content release by centrifugation (Figure 3). This solution provides a practical option for laboratories working with valuable samples or requiring post-collection procedures with minimal manual intervention.

This work aligns with a growing trend in which 3D printing has proven to be a valuable tool for addressing and improving experimental protocols in solid-state NMR. It enables the design of customized solutions tailored to specific needs. Within this context, the devices presented here expands the available repertoire with a particular focus on the efficient handling of semisolid samples in cryoprobe rotors, combining operational simplicity, reproducibility, and open accessibility for local fabrication and implementation.

## Conclusion

The increasing use of cryogenic probes in solid-state NMR studies has highlighted the need for specialized tools to handle samples in 90 μL rotors. In this work, a 3D-printed device was developed and validated for the efficient packing of semisolid samples into 3.2 mm rotors, optimizing sample transfer and minimizing manual handling. Its modular design, focused on operational simplicity, addresses limitations of conventional configurations and can be adapted to other formats. By providing the design files as an open-access resource, this device facilitates implementation across different laboratories and encourages the continued development of experimental solutions within a collaborative and evolving research environment.

## Supplementary Material

Supporting Information

zip file with files for 3D printing and disclaimer note
