# Supplementary material for: 3D-printed device for efficient packing of semisolid samples in 3.2 mm rotors used in cryoprobe systems
Source: Magn Reson Chem. Author manuscript; Available in PMC 2025 Oct 1. (PMC7617942; doi:10.1002/mrc.70010)
Supplement: Supporting Information [file EMS206664-supplement-Supporting_Information.pdf]

# Supporting Information for:

## 3D-printed device for efficient packing of semisolid samples in 3.2 mm rotors used in cryoprobe systems

Andrea Gelardo[1], Gustavo A. Titaux-Delgado[1*]

[1]Instituto de Química Física Blas Cabrera, Consejo Superior de Investigaciones Científicas (IQF-CSIC), Serrano 119, 28006 Madrid, Spain.

*Correspondence: gtitaux@iqf.csic.es

To facilitate the implementation of the proposed tools for sample handling, we provide the design files in STL format for 3D printing. Since a key aspect of device performance is the precise fit between the rotor and the internal cavity of the packing tool, minor deviations arising from the printing process such as material expansion or shrinkage depending on the resin used can affect this fit. To address this issue, we include four versions of the packing device, with internal cavity diameters of 3.27 mm, 3.28 mm, 3.29 mm, and 3.30 mm, respectively. These variations are introduced in 0.01 mm increments to allow users to test and select the version that provides the best fit for their specific printing conditions and materials.

Each version is labeled with a unique marking on the top surface of the device for easy identification, as shown in Supplementary **Figure S1**. This modular approach ensures broader compatibility and reproducibility across different laboratory environments and printing workflows.

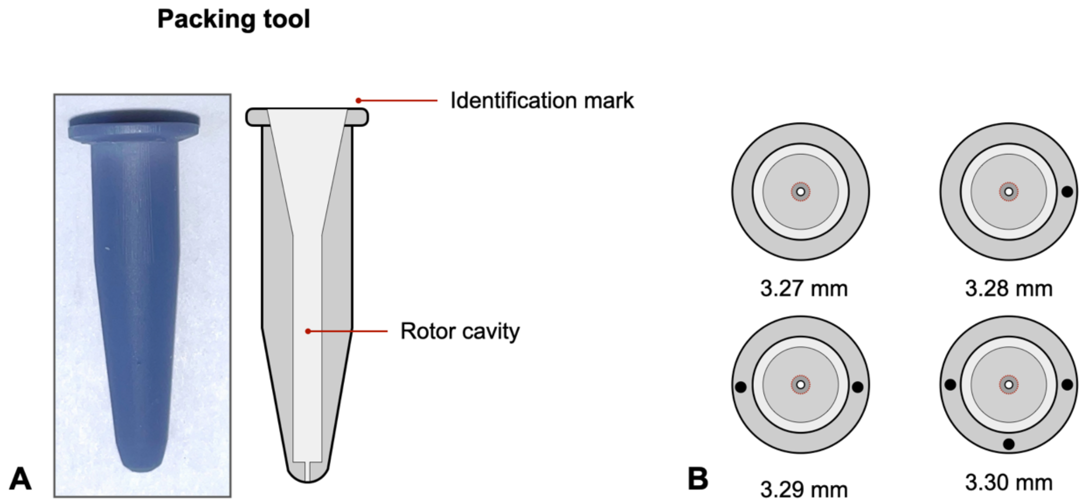


**Figure S1. Identification of packing device variants.** A. Shematic representation of the 3D-printed packing device designed for 3.2 mm rotors. B, View of the top surface of the device showing the engraved markings used to identify the internal cavity diameter where the rotor is inserted. Four variants are included: 3.27 mm (no marking), 3.28 mm (one mark), 3.29 mm (two marks), and 3.30 mm (three marks), respectively.
